# On the use of aspect-based sentiment analysis of Twitter data to explore the experiences of African Americans during COVID-19

**DOI:** 10.1038/s41598-023-37592-1

**Published:** 2023-07-02

**Authors:** Meghna Chaudhary, Kristin Kosyluk, Sylvia Thomas, Tempestt Neal

**Affiliations:** 1 Department of Computer Science and Engineering, University of South Florida, Tampa, FL USA; 2 Department of Mental Health Law and Policy, University of South Florida, Tampa, FL USA; 3 Department of Electrical Engineering, University of South Florida, Tampa, FL USA

**Keywords:** Computer science, Public health

## Abstract

According to data from the U.S. Center for Disease Control and Prevention, as of June 2020, a significant number of African Americans had been infected with the coronavirus disease, experiencing disproportionately higher death rates compared to other demographic groups. These disparities highlight the urgent need to examine the experiences, behaviors, and opinions of the African American population in relation to the COVID-19 pandemic. By understanding their unique challenges in navigating matters of health and well-being, we can work towards promoting health equity, eliminating disparities, and addressing persistent barriers to care. Since Twitter data has shown significant promise as a representation of human behavior and for opinion mining, this study leverages Twitter data published in 2020 to characterize the pandemic-related experiences of the United States’ African American population using aspect-based sentiment analysis. Sentiment analysis is a common task in natural language processing that identifies the emotional tone (i.e., positive, negative, or neutral) of a text sample. Aspect-based sentiment analysis increases the granularity of sentiment analysis by also extracting the aspect for which sentiment is expressed. We developed a machine learning pipeline consisting of image and language-based classification models to filter out tweets not related to COVID-19 and those unlikely published by African American Twitter subscribers, leading to an analysis of nearly 4 million tweets. Overall, our results show that the majority of tweets had a negative tone, and that the days with larger numbers of published tweets often coincided with major U.S. events related to the pandemic as suggested by major news headlines (e.g., vaccine rollout). We also show how word usage evolved throughout the year (e.g., *outbreak* to *pandemic* and *coronavirus* to *covid*). This work also points to important issues like food insecurity and vaccine hesitation, along with exposing semantic relationships between words, such as *covid* and *exhausted*. As such, this work furthers understanding of how the nationwide progression of the pandemic may have impacted the narratives of African American Twitter users.

## Introduction

Former United States (U.S.) surgeon general Jerome Adams highlighted the impact of the coronavirus pandemic on the African American population in 2020^1,2^. According to the U.S. Center for Disease Control and Prevention (CDC), over one-fifth (21.8%) of African Americans had been infected with the coronavirus disease as of June 2020^3^. U.S. states like Louisiana, Wisconsin, Michigan, Illinois, North Carolina, and New York also reported disproportionately higher death rates among African Americans due to COVID-19^4–7^, with higher percentages of death among African Americans under the age of 65 in comparison to European Americans/Whites (24.2% versus 9.7%)^8^. Prior studies have accredited these impacts to health disparities, or “systematic differences in health outcomes between groups and communities based on socioeconomic isolation”^9^. These include differences in income and higher rates of pre-existing health conditions, multi-generational housing, and comorbidity^1,9–14^. Many of these problems are long-standing and have had sustainable impacts^15^. For instance, higher cases of fatality and mortality rates were observed in the African American population during the 1918 Spanish influenza pandemic^16,17^, attributed to “understaffed and under-resourced”^18^ hospitals treating the African American population. This subsequently led to many relying on at-home care in “difficult living conditions driven by poverty, racism, and discrimination”^18^. Similarly, during the 2009 H1N1 influenza pandemic, African Americans faced increased susceptibility to health-related complications, such as coronary heart disease, obesity-related asthma, diabetes, and higher hospitalization rates^16,19,20^. It is important to understand the experiences, behaviors, and opinions of the African American population specific to the COVID-19 pandemic, especially as they relate to navigating matters of health and well-being, to encourage health equity, eliminate health disparities, and resolve sustained barriers to care.

Twitter data has shown significant promise as a representation of human behavior and for opinion mining, e.g., to measure well-being^21^, for income analysis^22^, to understand the emotional responses of Twitter users toward urban green spaces^23^, and as a proxy for human mobility^24^. Further, Twitter data has been widely used in other pandemic-related studies, such as to develop an early warning system of COVID-19 waves^25^, for sentiment and topic analysis of discussions surrounding COVID-19^25,26^, to monitor topic shifts in discussions from U.S. Twitter users before and after the emergence of COVID-19^27^, and to extract people’s opinions of COVID-19 vaccines^28^. In this study, we also leverage the ubiquitous nature of social media data via the Twitter platform to characterize the pandemic-related experiences of the African American community using aspect-based sentiment analysis on tweets published in 2020.

Sentiment analysis is a common task in natural language processing (NLP) that identifies the emotional tone, or polarity, (i.e., positive, negative, or neutral) of a piece of text. Aspect-based sentiment analysis increases the granularity of sentiment analysis by also extracting the aspect (i.e., an entity, target, or feature) for which sentiment has been expressed^29,30^. For example, in the statement, “the food is delicious and the ambience of this restaurant is good,” the overall sentiment is *positive*. However, in aspect-based sentiment analysis, a *positive* sentiment is expressed toward the aspects of *food* and *ambience* with the associated opinion terms of *delicious* and *good*, respectively. Using this methodology, we seek to identify various aspect terms found in tweets related to COVID-19 with positive, negative, or neutral polarity. We also highlight certain aspect terms used in tweets that were published around the same time as major U.S. events in 2020, and examine how the use of these aspect terms may have changed over time (e.g., the use of the term *outbreak* decreased early in the year as *pandemic* began to increase in March). By doing so, we aim to further understanding of how the nationwide progression of the pandemic may have impacted the narratives of African American Twitter users.

## Related work

Researchers have begun examining the relationship between race and/or ethnicity with COVID-19 outcomes. For instance, Abuelgasim et al.^31^ highlighted risks that are more likely to lead to severe COVID-19 diseases posed by existing health conditions, such as decreased lung function and higher rates of cardiovascular disease in ethnic minorities^32^. Tai et al.^32^ noted that, in particular, African Americans have a disproportionately higher prevalence of such comorbidities, including diabetes, hypertension, obesity, and coronary artery disease, contributing to disproportionate deaths among African Americans with COVID-19. However, the authors also pointed out another important issue faced by ethnic minorities—that is, “before the pandemic and associated economic fallout, poverty rates in the United States were 24% for Native Americans, 22% for African Americans, and 19% for Hispanics, compared to 9% for Whites”^32^. The authors further elaborated on such economic inequalities, noting that larger percentages of minorities are financially disadvantaged, have fewer flexible work options (e.g., work from home), and experience higher likelihoods of exposure to “occupational hazards” like commuting via public transportation.

With such increased risk, others have taken a closer look at vaccination uptake among U.S. minorities as a protective measure. Carson et al.^33^ conducted a series of focus groups to assess views toward vaccines, wherein participants stated concerns arising from conflicting and questionable vaccine information that circulated in the news, prior vaccines that were experimental and discriminatory, vaccine accessibility, language barriers, occupational barriers (such as lack of transportation or paid time off), and lack of health insurance. Winifred et al.^34^ conducted a similar focus group-based study to assess views on vaccine trials at hospitals, where their participants worried that they might contract COVID-19 while being vaccinated or have adverse side effects. Participants also voiced distrust for vaccination, noting “hidden agendas” and “cultural appropriateness”^34^. Hildreth and Alcendor^35^ reiterated the relationship between this distrust and vaccine hesitancy, noting that some African Americans’ belief that the COVID-19 vaccines were rushed in their development might be fueled by the lingering impacts of “social, political, and economic injustices”^35^. They called for health equity initiatives to address these problems. However, the undersupply of research on the African American community might further prolong the impacts of such inequities as missing knowledge persists. The current study aims to fill some of these gaps via content analysis of Twitter data. Twitter analyses facilitate the gathering of a worldwide pool of discourse on a topic of interest, leading to understanding human experiences in ways that reflect peoples’ attitudes in their natural settings^25,26,36^.

Although, to our knowledge, no prior work has applied aspect-based sentiment analysis to Twitter data to study conversational patterns surrounding COVID-19 within the U.S. African American population, some researchers have applied NLP techniques to Twitter data (e.g., sentiment analysis or topic modeling) to study attitudes and perceptions related to COVID-19. Odlum et al.^37^ applied topic modeling to tweets which used hashtags #blacktwitter, #staywoke, and #blacklivesmatter as a representative dataset of tweets produced within the African American Twitter user base. They extracted *n*-grams from approximately 2.6 million tweets published from January to May 2020. Four themes emerged related to COVID-19, including symptoms and transmission patterns, treatment and cures, interventions, and fear (i.e., protection, isolation, food shortage, etc.). We note, however, that this work is the only identified with specific focus on the African American community.

In related work, Kleinberg et al.^38^ collected 5000 written responses, including short texts generalizable to tweets, from 2500 participants in a survey which focused on the mental and emotional impacts of COVID-19 in the UK. Topic modeling was applied, identifying home, work, family, economy, employment, and lockdown as important. They also found anxiety, worry, sadness, and fear as dominant self-reported states. Similarly, Cheng et al.^25^ also leveraged NLP to prototype a country-specific early warning system that predicts new COVID-19 cases in the UK according to the total volume of tweets to assist policymakers. Topic modeling and sentiment analysis were applied; key topics of discussion included cases, deaths, support or help, the UK government, retail, and mask, among others. They also found positive sentiment expressed toward the topics of testing, tracing, vaccines, and face masks, with spikes in sentiment correlating with real-world events like the time frame of mask shortages. Other studies also investigate NLP, particularly using topic and sentiment analysis, on data concerning COVID-19. Zhang et al.^36^ correlated real-world events with peaks in the number of positive COVID-19 cases using Twitter data collected between January 20 and May 15, 2020, finding oil/stock prices, herd immunity, working/studying from home, economic stimulus, medicine/vaccines, and employment as important topics, positive sentiment toward working and studying from home, and denial concerning herd immunity. Xue et al.^26^ found lockdown, staying at home, new cases, confirmed cases, death toll, public health measures, social stigma, quarantine, and social distancing as dominant topics.

## Methodology

Our analyses concentrate on the period when COVID-19 first started affecting the United States, approximately January 2020. Given that COVID-19 was a novel disease, managing the swift progression into a pandemic was a new global challenge. Examining this specific year offers a comprehensive understanding of emerging viewpoints, behavioral patterns, and evolving opinions. Thus, this study required a dataset of tweets related to COVID-19 published in 2020 on which aspect-based sentiment analysis could be applied. To this end, we used a collection of tweet IDs compiled by Chen et al.^39^. These IDs provide access to over a billion tweets that were originally published from January 21, 2020 through December 31, 2020 in English by Twitter users with profiles geo-tagged in U.S. states. The tweet IDs were collected using the Twitter streaming application programming interface^40^ and Tweepy^41^. Chen et al.^39^ collected these data using 80 keywords, such as ‘Sars-cov-2’, ‘staysafestayhome’, ‘Coronials’, ‘Covid’, ‘pandemic’, and ‘Covid19’ to develop a repository associated with COVID-19. However, some keywords like ‘cdc’ and ‘china’ could lead to irrelevant tweets as they reflect multiple contexts. Further, since tweet objects (which contain fields such as text, creation time, profile image URLs, etc.)^42^ retrieved using tweet IDs do not contain information about the demographic data of the user, it is also necessary to develop methods to do so. Thus, our methodology reflects a multi-step process to discard tweets that are not related to COVID-19 and tweets that are not likely to have been authored by African American Twitter users.

### Classifying tweets related to COVID-19

An initial challenge for this analysis was the presence of noise—that is, tweets that are unrelated to COVID-19. For example, tweets retrieved using the keyword ‘corona’ included those referring to COVID-19 as well as Corona beer. The keyword ‘pandemic’ included tweets on both the COVID-19 and vaping pandemics^43^. Similarly, the use of the keyword ‘china’ yielded tweets not only about COVID-19, but also those on general news about China. To discard irrelevant tweets, we trained and evaluated several machine learning classifiers to classify a tweet as either related or not related to COVID-19.

Using the Twitter COVID-19 stream^44^, 27,068 tweets from 280 tweet files published from November 6, 2020 to December 6, 2020 were randomly acquired using Python’s random.py function as positive examples (i.e., tweets relevant to COVID-19). Similarly, an additional 27,068 tweets from the Twitter Academic Track Archive^45^ published from October 2019 to November 2019 were acquired as negative examples (i.e., tweets not relevant to COVID-19 since the first COVID-19 case was not reported until December 2019). We note that the negative examples were selected using the same keywords as proposed by Chen et al.^39^ to allow the model to learn from examples that may use similar vocabulary (e.g., using words like ‘china’ or ‘corona’), yet are not related to COVID-19. This was facilitated by leveraging the ability to query using more keywords and streaming rules (1024 characters, 1000 streaming rules) via the Twitter Academic Track Archive, contrasting what would have been capable by using the standard Twitter streaming API. All tweets were written in English, geo-tagged in the U.S., and pre-processed by removing hyperlinks, mentions, stopwords, and hashtag signs (but not the hashtag text), and converting emojis to their corresponding textual equivalent. Together, these tweets were randomly split such that 75% were used as training data and 25% as validation data. In total, the dataset consisted of 40,602 training and 13,534 validation examples to investigate the use of machine learning classifiers to automate the labeling of a tweet as related or not related to COVID-19. The validation data was used for model fine-tuning.

As test data, we utilized the free-form responses from a COVID-19 related survey conducted by our research team. The purpose of the survey was to gain insights into how COVID-19 impacted individuals from various racial and ethnic backgrounds, as well as their willingness to seek help, barriers to healthcare, income, access to health insurance, and their opinions on public news related to COVID-19. The survey was conducted from February 2021 to March 2022 and consisted of 11 questions requiring free-form responses. In total, we received 82 responses, comprising 59 male, 21 female, and 2 gender variant participants. The racial distribution of the respondents was as follows: 30 African African/Black, 26 White, 26 Hispanic, Latino, or Spanish, 2 American Indian/Alaskan Native, 12 Non-resident Alien, 7 Asian, and 1 participant identifying as two or more races. While acknowledging that this survey captured data representing a different time span (2021–2022) from the time frame of this study (2020), its focus on COVID-19 renders it a valuable complement to our research. Therefore, we utilized the survey responses as positive test examples. These survey questions are provided in Appendix A.1. It is important to note that our survey received Human Subjects Approval from our Institutional Review Board (Study #STUDY002133).

To generate negative test examples, we employed two sources. First, we randomly selected 1984 tweets from the Twitter Academic Track Archive^45^. Additionally, we collected 770 negative test examples provided by Ardehaly and Culotta^46^. It is worth noting that these tweets were published when Twitter allowed a maximum of 140 characters per tweet, whereas the current limit is 280 characters. By incorporating tweets with shorter lengths, we aimed to evaluate a classifier’s ability to discern the distinguishing characteristics of each class while varying the available information.

The test examples were divided into two test sets. The first test set consisted of 720 positive test survey examples alongside the 1984 tweets from the Academic Track Archive as negative test examples. For the second test set, we used the same positive examples as in the first set, but the negative examples comprised the 770 140-character tweets.

We evaluated four classification models, including a Logistic Regression model, the Bidirectional Encoder Representations from Transformers (BERT) model, a linear Support Vector Machine (SVM), and Multinomial Naive Bayes, to classify data as related or not related to COVID-19. Logistic regression fits a sigmoid function ranging from zero to one to predict the probability of a dichotomic dependent variable occurring from one or more independent variables^47^; the model’s output above 0.5 is the distinguishing threshold for separating two classes. BERT is a language model that randomly masks input words to make context-based predictions^48^. A linear SVM computes an optimal hyperplane which can linearly separate data samples; the hyperplane is chosen such that the distance, or margin, between the hyperplane and closest data samples (referred to as support vectors) is maximized^49^. Finally, in Multinomial Naive Bayes, a text sample is represented as an ordered set of words from its vocabulary according to a multinomial distribution of words^50^.

With the exception of the BERT classifier, each classifier was trained and evaluated on 30,000 unigrams, bigrams, and trigrams features that were extracted from the data. Classification accuracy was assessed using the $F_1$ score, or the harmonic mean of precision (*P*) and recall (*R*):

$$\begin{aligned}F_1 = \frac{2 * P * R}{P + R} \mathrm {,\, where } \end{aligned}$$

$$\begin{aligned}P = \frac{\mathrm {\#\, of \,True\, Positives}}{\mathrm {\#\,of\,True\,Positives} + \mathrm {\#\,of\, False \,Positives}} \mathrm { \,and\, } \end{aligned}$$

$$\begin{aligned}R = \frac{\mathrm {\# \,of \,True\, Positives}}{\mathrm {\# \,of \,True\, Positives} + \mathrm {\# \,of \,False \,Negatives}}. \end{aligned}$$

The BERT model demonstrated a slight superiority over all other models, achieving a macro-average $F_1$ score of 92% in predicting the relevance of tweets to COVID-19 in the first test set. However, all models performed slightly below chance (less than 50%), except for Multinomial Naive Bayes, which attained a macro-average $F_1$ score of 63% in the second test set. We attributed this observation to the relatively shorter text lengths among the  negative examples. Consequently, we utilized the Multinomial Naive Bayes classifier to distinguish COVID-19 tweets from non-COVID-19 tweets.

### Demographic classification of COVID-19 tweets

In the second phase of our filtering process, we aimed to train a classifier to predict the racial background of individuals who posted COVID-19-related tweets. The objective was to identify and retain tweets likely authored by African American Twitter users. However, tweet objects themselves do not provide demographic data, which prompted the need for alternative approaches. Previous studies have employed methods such as mapping census data to geo-tags or utilizing human annotators to label tweets based on the author’s race^46,51–54^. However, relying on census statistics can result in outdated information, geo-tags may not always be available or accurately represent the author’s home or permanent location, and human annotation introduces the potential for bias^55^.

Our approach involved training two models—a combination of an image classifier and a language-based classifier—to leverage both visual and linguistic cues of one’s racial identity. This dual-model approach became necessary due to various challenges encountered in the data. For instance, many Twitter profile image URLs were non-functional or corrupt, some downloaded images were also corrupt, and functional URLs did not always contain images of real people (e.g., cartoon characters, animals, etc.). Consequently, we found image classification alone to be impractical. However, existing research has indicated that language patterns can be influenced by ethnicity. Distinct dialects, references, contractions, frequent use of native words, and specific phonetic choices have been observed among individuals of different racial groups^56,57^.

**Figure 1:**
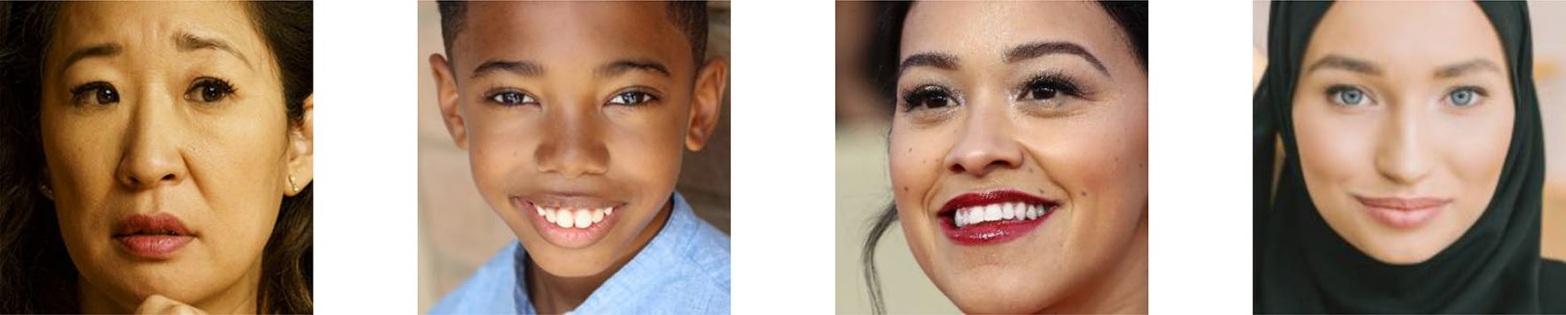
Examples of face images extracted from the FairFace dataset^61^, showcasing individuals from different racial categories (Asian, Black, Latino, and Middle Eastern from left to right).

First, we obtained a subset of images from the publicly available dataset called FairFace^58^. FairFace consists of 108,501 face images, each labeled with one of the following categories: White, Black, Indian, East Asian, Southeast Asian, Middle East, or Latino. We selected all 12,233 images labeled as Black from FairFace as positive examples. For negative examples, we randomly chose an equal number (2039) of images from each of the other categories. Figure 1 provides examples of the images extracted from FairFace. To classify the race of individuals depicted in the images, we trained a Convolutional Neural Network (CNN)^59,60^. CNNs are widely used in deep learning for image-based classification tasks. They consist of multiple layers of neurons designed to detect progressively more complex features, starting with simpler features like edges and gradually learning higher-level, abstract features.

**Figure 2:**
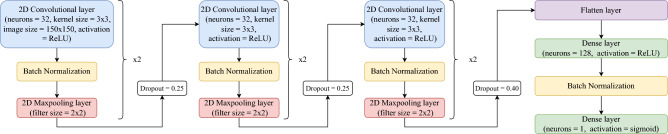
Architecture of the convolutional neural network (CNN) model used for race classification.

For our CNN model, we employed an Adam optimizer with a learning rate of 0.001. We incorporated batch normalization between layers, dropout, and data augmentation techniques to enhance the model’s performance. The architecture of our CNN model is illustrated in Fig. 2. To assess the performance of the model, we divided the images into training data (75%) and validation data (25%). Using this setup, we achieved an 88% macro-average $F_1$ score for race classification. Exact classifier parameters and all code developed for this project are publicly accessible at github.com/nlp-grp/AfricanAmericans_COVID19_Perceptions.

We conducted testing of the trained CNN using two publicly available datasets: UTKFace^62^ and FaceARG^63^. The UTKFace dataset consists of over 20,000 face images with dimensions of $128\times 128$. These images are annotated with labels for race (White, Black, Asian, Indian, and other), gender, and age. Specifically, the racial distribution in UTKFace includes White (10,352), Black (4636), Asian (3527), Indian (23,707), and other (Hispanic, Latino, Middle Eastern) (1741) individuals. The FaceARG dataset comprises more than 175,000 images with dimensions of $299 \times 299$. These images were collected from the internet and are labeled with one of the following races: African American (24.02%), Asian (25.60%), Caucasian (24.42%), or Indian (25.94%).

To assess the generalization capability of the CNN model on unseen examples, we tested it on the entire UTKFace dataset and a subset of 42,000 images from the FaceARG dataset. This subset consisted of 21,000 images from the African American class and 21,000 images from the other racial classes. The CNN model achieved an 85% macro-average $F_1$ score on the FaceARG dataset and an 89% macro-average $F_1$ score on the UTKFace dataset. These results indicate that the model was sufficiently trained to serve as an image-based race classifier as the $F_1$ scores align with existing literature^64^. The classification reports generated by Scikit-Learn for each dataset are provided in Table 1.

**Table 1:** Classification reports for the FairFace and UTKFace datasets, showing performance metrics (precision, recall, $F_1$-score, and support) for each racial class.

		FairFace dataset				UTKFace dataset			
		Precision	Recall	$F_1$ Score	Support	Precision	Recall	$F_1$ Score	Support
Class	Other races	0.80	0.94	0.86	21,000	0.97	0.97	0.97	28,557
	African American	0.92	0.76	0.83	21,000	0.81	0.80	0.81	4,931
Accuracy				0.85	42,000			0.94	33,488
Macro average		0.86	0.85	0.85	42,000	0.89	0.88	0.89	33,488
Weighted average		0.86	0.85	0.85	42,000	0.94	0.94	0.94	33,488

Finally, as mentioned, it is important to note that the image classifier is not sufficient on its own for our purposes. A large percentage of profile images are non-human (e.g., cartoons or animals), pictures of celebrities, corrupt, etc. However, all tweets have corresponding text data (i.e., the tweets themselves), such that a language-based classifier, or a classifier trained on the content of the tweets themselves instead of relying on a profile pictures, is better able to generalize for the purpose of classifying one’s race. Thus, we applied the trained image classifier to the profile images associated with user profiles gathered from the COVID-19 stream^44^. These 50,000 profiles were those remaining after extracting image URLs from Twitter objects for more than 2.3 million URLs after filtering out non-functional URLs, corrupt images, non-human images, celebrity images, and images labelled as containing human faces by Microsoft Azure Cognitive Services with less than 50% confidence. We used the image classifier to label these images by race. The number of images obtained for the African American class were 3411. We randomly selected an equal number of images from the Other Races class leading to total 6822 images. Afterward, we annotated the corresponding tweets’ texts with the same labels. We, then, trained a language-based model using these race-labelled tweets by evaluating both machine learning (Multinomial Naïve Bayes, linear Support Vector Machine, and Logistic Regression) and deep learning models (including BERT, LSTM^65^, BiLSTM^66^, BiGRU^67,68^, BiGRU-CNN^69^) for race classification. Since unigrams have been observed to correlate with demographic data, including race, as they capture idiosyncratic words and spellings, we extracted unigrams, in addition to part-of-speech tags, as features for training^57^. Specifically, we extracted 10,000 unigrams from the COVID-19 related tweets now labeled by race according to the subscriber’s profile image using the image classifier.

**Figure 3:**
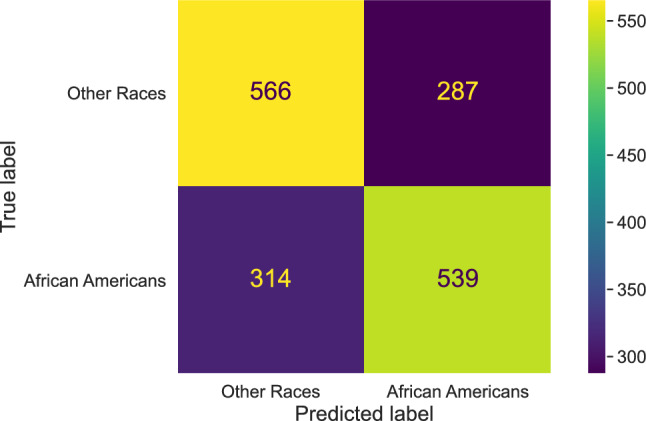
Confusion matrix for when using a Naive Bayes classifier to develop a language-based race classification model.

The non-linear, deep models showed model overfitting when evaluating against validation data. However, among the machine learning models, the Multinomial Naïve Bayes outperformed the other models, consistent with other studies. For example, a Naïve Bayes classifier has been used for authorship classification of tweets^70^, for identification of disaster-related informative tweets^71,72^, identifying tweets with hate content^73^, classifying tweets into topic-based categories^74^, and for sentiment analysis of COVID-19 tweets^75^. In our work, the Naïve Bayes classifier resulted in 61% training accuracy and 65% test accuracy, with a 65% macro-average $F_1$-score. The confusion matrix is given in Fig. 3.

**Figure 4:**
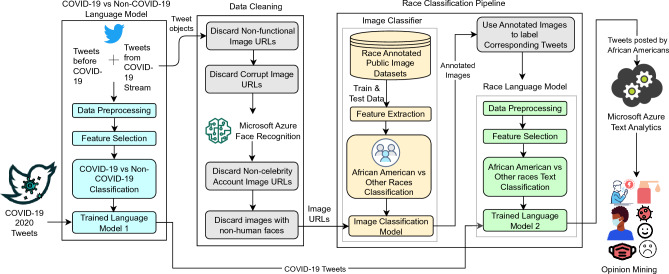
Experimental methodology framework.

### Opinion mining with aspect-based sentiment analysis

Microsoft Azure Cognitive Services provides sentiment analysis and opinion mining software as a part of its cloud computing platform, which we leveraged in this work^76^. Azure’s sentiment analysis outputs three labels, positive, negative, and neutral, along with the confidence score (0 to 1 indicating low to high confidence) of the predicted sentiment label, for a given text sample. Opinion mining, on the other hand, provides more granular information in terms of identifying the target (aspect) for which the opinion has been expressed, the expressed opinion, and the sentiment. We configured each resource for the English language and to run synchronously (that is, outputting results immediately without intermediate data storage). The entire data filtering and model architecture is provided in Fig. 4.

## Results

Our data filtering process, as detailed in Section “Methodology”, consists of multiple steps to remove tweets that are unlikely to be related to COVID-19 and unlikely to have been published by a Twitter user which identifies as African American and/or Black. We applied this filtering framework to tweets extracted from over a billion tweet IDs collected by Chen et al.^39^. Specifically, for the more than a billion tweets publicly available via Chen et al., we first removed those classified as not related to COVID-19 using the Naive Bayes model described in Section “Classifying tweets related to COVID-19”. Then, we applied the language-based classifier to the tweet contents of the remaining COVID-19 related tweets to retain only those likely to have been authored by African American Twitter subscribers. After completing all data filtering steps, 3,955,729 tweets remained to investigate aspect-based sentiments.

### Tweet frequency and polarity

**Figure 5:**
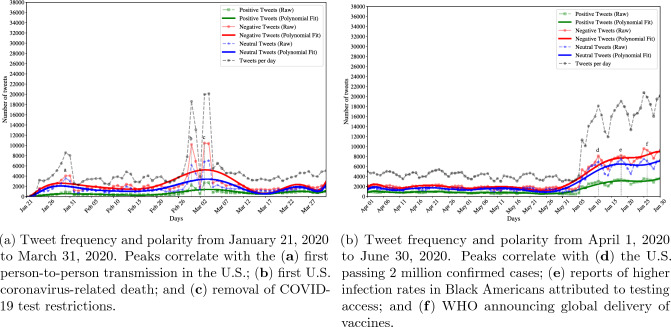
Trends in tweet frequency and polarity (positive, neutral, or neutral) from January to June. Peaks (significant increases in the number of tweets published on a specific date within a span of time) are denoted and correlated with events reported in national news headlines^77–79^. The gray line indicates the total number of tweets per day.

**Figure 6:**
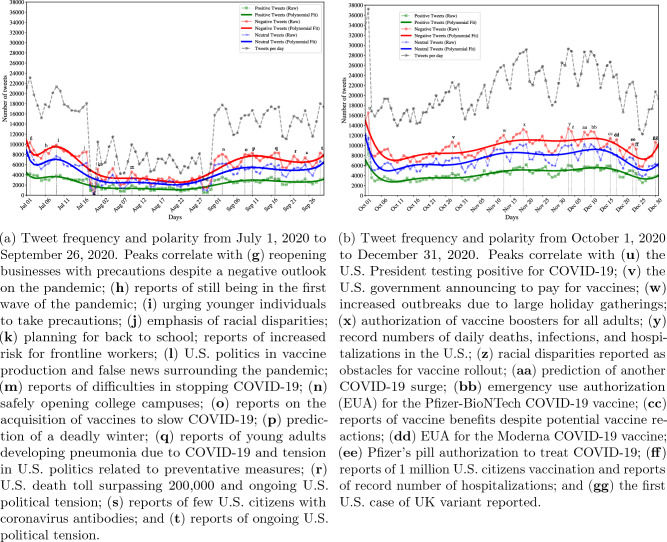
Trends in tweet frequency and polarity (positive, neutral, or neutral) from July to December. Peaks (significant increases in the number of tweets published on a specific date within a span of time) are denoted and correlated with events reported in national news headlines^77–79^. The gray line indicates the total number of tweets per day.

Figures 5 and 6 depict the distribution of positive, negative, and neutral tweets that were retained for analysis. Each figure displays two line graphs: the thinner line represents the actual number of daily tweets (raw counts), while the thicker line represents the overall trend of tweet frequency derived from fitting a polynomial function of degree 10 to the raw counts. These visualizations reveal that the majority of tweets exhibit a negative polarity, with increased Twitter activity observed from early March through June and July, as well as during the last quarter of the year.

The observed trends in Twitter activity align with significant developments related to the onset of the pandemic. Notably, the peak observed in early March (Fig. 5a,b) coincides with the first reported death due to COVID-19 in the United States. In contrast, the more subtle increase (Fig. 5a,a) in Twitter activity occurred around the time of the first confirmed person-to-person transmission in late January. It is important to note that there were considerably fewer tweets discussing the virus until March, despite public awareness of its existence. This discrepancy may be attributed to major news headlines regarding the spread of misinformation, which potentially influenced the perceptions of some African Americans and resulted in decreased concern and fewer discussions about the virus on Twitter. For instance, reports highlighted messages suggesting that the virus would dissipate in spring with warmer weather or that African Americans possessed a special immunity or resistance to COVID-19^80^. Notably, it was reported that the “Black community [had] been specifically targeted by misinformation surrounding the coronavirus”^81^. We hypothesize that the first death drew more attention to the severity of the virus, discredited misinformation, and consequently generated increased discussion. This is supported by the ratio of negative to positive tweets between peaks **a** and **b**. On January 21, there were approximately 3500 more negative tweets than positive tweets, while by March 1, there were 8000 more negative tweets than positive tweets. Overall, the number of negative tweets spiked significantly above 10,000 from peaks **a** to **b**, representing a nearly 233% increase in negative discourse on Twitter. This indicates a growing sense of concern, worry, disbelief, or other unfavorable emotions as the pandemic escalated throughout the first quarter of the year.

Figure 5b illustrates the distribution of positive, negative, and neutral tweets during the second quarter of 2020. One notable observation is the increase in published tweets throughout the month of June. Interestingly, unlike the predominantly negative discourse observed in peaks **b** and **c** in Fig. 5a, peaks **d**, **e**, and **f** display a mixture of negative and neutral tweets. This could indicate mixed emotions, such as heightened distress alongside indifference, towards events occurring in June. These events include the milestone of total COVID-19 cases surpassing two million (**d**), reports indicating unequal access to COVID-19 testing for African Americans leading to higher infection rates (**e**), and the World Health Organization (WHO) announcing global vaccine distribution (**f**). It is also possible that the neutral tweets reflect the dissemination of information or news about the pandemic in an impartial manner. Notably, the term *news* emerged as one of the top 10 most frequently used aspect terms when analyzing the frequent aspects for June.

Figure 6a displays the frequency and polarity of tweets from July 1, 2020 to September 30, 2020, showing an overall increase in published tweets during the latter part of the year. According to the information outlined in Table 2, the third quarter of the year was marked by nationwide attention toward the worsening of the pandemic, discussions about returning to a sense of “normalcy” in the upcoming months (such as reopening businesses, schools, and college campuses), reports on vaccine delivery, and political tensions within the U.S. government. Moving towards the end of the year, Fig. 6b shows sustained high levels of tweet activity. Once again, the majority of peak events align with vaccine discussions, indicating that the African American community may have been particularly focused on the vaccine rollout. Notably, during December 2020, several news articles were published highlighting the “deep distrust of potential vaccine efforts”^82^ (e.g.,^83–85^), potentially explaining the higher rates of tweet publication and an increased proportion of negative tweets. A comprehensive summary of all peak events throughout 2020, along with corresponding news headlines, can be found in Table 2.

**Table 2:** List and dates of major news headlines in 2020 related to the coronavirus pandemic that occurred around the same time as significant increases in the number of tweets posted on or near the same date. “L” (0–6000), “M” (6000–12,000), and “H” (12,000+) refer to low, medium and higher numbers of positive, negative, and neutral tweets.

Date	Headline	Pos.	Neu.	Neg.
Jan. 30	First U.S. case of person-to-person transmission^79^	L	L	L
Feb. 29	First coronavirus-related U.S. death^79^	L	M	M
Mar. 3	Removal of test restrictions^79^	L	M	M
Jun. 11	U.S. passes 2 million confirmed cases^79^	L	M	M
Jun. 18	Higher infection rates in Black Americans attributed to testing access^78^	L	M	M
Jun. 26	WHO announces global delivery of vaccines^79^	L	M	M
Jul. 2	Considerations of reopening businesses with precautions are announced despite a negative outlook on the pandemic^78^	L	M	M
Jul. 6	Experts report still being in the first wave of the pandemic^78^	L	M	M
Jul. 9	Younger individuals urged to practice precautionary measures^78^	L	M	M
Jul. 16	Racial disparities emphasized by experts^78^	L	M	M
Aug. 1	Experts recommend planning for back to school; reports of increased risk for frontline workers^78^	L	L	L
Aug. 5	U.S. politics highlighted in vaccine production and false news surrounding the pandemic^78^	L	L	L
Aug. 10	Experts report difficulties in stopping COVID-19^78^	L	L	L
Sept. 3	Experts discuss safely opening college campuses^78^	L	M	M
Sept. 9	Experts report on the acquisition of vaccines to slow COVID-19^78^	L	L	M
Sept. 11	A deadly winter is predicted^78^	L	L	M
Sept. 17	Reports of young adults developing pneumonia due to COVID-19 and tension in U.S. politics related to preventative measures^78^	L	L	M
Sept. 22	U.S. death toll surpasses 200,000 and ongoing U.S. political tension related to the pandemic^77,78^	L	L	M
Sept. 25	Reports of few U.S. citizens with coronavirus antibodies; U.S. reported to still be in the first wave^78^	L	M	M
Sept. 29	Reports of ongoing U.S. political tension regarding preparation for a potential pandemic^78^	L	M	M
Oct. 2	U.S. President tests positive for the coronavirus^77^	M	M	H
Oct. 28	U.S. government announces to pay for future coronavirus vaccines^78^	L	M	M
Nov. 13	Increased outbreaks reported due to large holiday gatherings^77^	L	M	H
Nov. 19	The FDA authorizes vaccine boosters for all adults^78^	L	M	H
Dec. 3	U.S. sets records of daily deaths, infections, and hospitalizations^78^	L	M	H
Dec. 4	Racial disparities reported as obstacles for vaccine rollout^78^	L	M	H
Dec. 8	Another COVID-19 surge predicted after holiday season^78^	L	M	H
Dec. 11	FDA issues Emergency Use Authorization for the Pfizer-BioNTech COVID-19 vaccine^77^	L	M	H
Dec. 16	Reports reassure benefits of vaccines despite potential vaccine reactions^78^	L	M	M
Dec. 18	FDA issues Emergency Use Authorization for the Moderna COVID-19 vaccine^77^	L	M	M
Dec. 23	FDA authorizes Pfizer’s pill to treat COVID-19^78^	L	M	M
Dec. 24	More than 1 million U.S. citizens are vaccinated; U.S. reports record number of hospitalizations^77,78^	L	M	M
Dec. 30	First U.S. case of UK variant reported^77^	L	M	M

### Frequent aspect terms

**Table 3:** Top aspect terms for peaks observed from January 2020 to June 2020.

First quarter			Second quarter		
Jan. 30: First U.S. case of person-to-person transmission	Feb. 29: First coronavirus-related U.S. death	Mar. 03: Removal of test restrictions	Jun. 11: U.S. passes 2 million confirmed cases	Jun. 18: Higher infection rates in Black Americans attributed to testing access	Jun. 26: WHO announces global delivery of vaccines
Coronavirus	Coronavirus	Coronavirus	Covid	Covid	Covid
China	Race	Corona	Coronavirus	News	Information
News	News	Thread	Pandemic	Coronavirus	Music
Corona	Corona	Corona virus	Spreads	Mask	Pandemic
Flights	Prevention	News	News	Pandemic	Mask
Passengers	Signature	Information	Masks	Cases	Coronavirus
Experts	Corona virus	Shipping	Mask	Food	Court
Information	Masks	Covid	Corona	Masks	News
Video	Patient	China	Cases	Place	Care act
Agencies	Information	Case	Service	Staff	Cases

**Table 4:** Top aspect terms for peaks observed from July 2020 to August 2020.

Third quarter						
Jul. 2: Considerations of reopening businesses with precautions are announced despite a negative outlook on the pandemic	Jul. 6: Experts report still being in the first wave of the pandemic	Jul. 9: Younger individuals urged to practice precautionary measures	Jul. 16: Racial disparities emphasized by experts	Aug. 1: Experts recommend planning for back to school; reports of increased risk for frontline workers	Aug. 5: U.S. politics highlighted in vaccine production and false news surrounding the pandemic	Aug. 10: Experts report difficulties in stopping COVID-19
Mask	Mask	Mask	Mask	Covid	Covid	Mask
Covid	Covid	Covid	Covid	Mask	Pandemic	Pandemic
Masks	News	News	Masks	Coronavirus	Mask	News
Pandemic	Masks	Staff	Pandemic	Pandemic	News	Covid
News	Pandemic	Pandemic	News	Masks	Food	Masks
Records	Virus	Masks	Finger	Doctor	Thread	Test results
Thread	Actor	Coronavirus	Coronavirus	Face	Corona	Staff
Face	Coronavirus	Place	Video	News	Interview	People
Cases	Corona	People	People	Corona	Cases	Thread
Coronavirus	Face	Churches	Place	People	Staff	Food

**Table 5:** Top aspect terms for peaks observed for the month of September 2020.

Third quarter (continued)						
Sept. 3: Experts discuss safely opening college campuses	Sept. 9: Experts report on the acquisition of vaccines to slow COVID-19	Sept. 11: A deadly winter is predicted	Sept. 17: Reports of young adults developing pneumonia due to COVID-19 and tension in U.S. politics related to preventative measures	Sept. 22: U.S. death toll surpasses 200,000 and ongoing U.S. political tension related to the pandemic	Sept. 25: Reports of few U.S. citizens with coronavirus antibodies; U.S. reported to still be in the first wave	Sept. 29: Reports of ongoing U.S. political tension regarding preparation for a potential pandemic
Covid	Covid	Covid	Intrusion	Covid	Covid	Covid
Mask	Pandemic	Pandemic	Revelation	Pandemic	Pandemic	Pandemic
Pandemic	News	Mask	Covid	News	Mask	News
News	Mask	News	Staff	Mask	News	Coronavirus
Equipment	Coronavirus	Food	Mask	Equipment	Statement	Tests
Claims	Equipment	Coronavirus	Emails	Masks	Coronavirus	Mask
Vaccine	Food	Masks	Pandemic	Coronavirus	Governor	Place
Coronavirus	Vaccine	Event	Woman	Billboard music awards	Masks	Cases
Story	Book	Game	Masks	Food	Food	Food
Staff	Article	Travel industry	News	Story	Staff	Rate

**Table 6:** Top aspect terms for peaks observed from October 2020 to December 04, 2020.

Fourth quarter					
Oct. 2: U.S. President tests positive for the coronavirus	Oct. 28: U.S. government announces to pay for future coronavirus vaccines	Nov. 13: Increased outbreaks reported due to large holiday gatherings	Nov. 19: The FDA authorizes vaccine boosters for all adults	Dec. 3: U.S. sets records of daily deaths, infections, and hospitalizations	Dec. 4: Racial disparities reported as obstacles for vaccine rollout
Mask	Covid	Covid	Covid	Covid	Covid
News	Pandemic	Mask	Mask	Court	Mask
Covid	Mask	Mask	Pandemic	Pandemic	Pandemic
Recovery	News	Pandemic	News	Mask	News
People	Justin turner	Court	Masks	News	Thread
Masks	Hospitals	News	Vaccine	Vaccine	Vaccine
Court	Masks	Thread	Outbreaks	Doctor	Masks
Pandemic	Cognitive costs	Court justice	Staff	Staff	People
Virus	Game	Woman	Coronavirus	Place	Pfizer
Song	Turner	Cases	Face	Leadership	Story

**Table 7:** Top aspect terms for peaks observed from December 05, 2020 to December 31, 2020.

Fourth quarter (continued)						
Dec. 8: Another COVID-19 surge predicted after holiday season	Dec. 11: FDA issues Emergency Use Authorization for the Pfizer-BioNTech COVID19 vaccine	Dec. 16: Reports reassure benefits of vaccines despite potential vaccine reactions	Dec. 18: FDA issues Emergency Use Authorization for the Moderna COVID-19 vaccine	Dec. 23: FDA authorizes Pfizer’s pill to treat COVID-19	Dec. 24: More than 1 million U.S. citizens are vaccinated; U.S. reports record number of hospitalizations	Dec. 30: First U.S. case of UK variant reported
Covid	Covid	Covid	Covid	Bill	Covid	Covid
Vaccine	Vaccine	Pandemic	Vaccine	Covid	Mask	News
Mask	Mask	Vaccine	Pandemic	Pandemic	Pandemic	Pandemic
News	News	Crew	Thread	News	News	Mask
Thread	Pandemic	News	News	Thread	Bill	Vaccine
Pandemic	Food	Staff	Mask	Mask	Story	Nurse
Pfizer vaccine	People	Thread	Court	Package	People	Story
Story	Mask	Finger	Staff	Staff	Food	Masks
Game	Story	Mask	Masks	Story	Republicans	Food
Face	Heart	Story	Food	Coronavirus	Vaccine	Face

Tables 3 through 7 present the top 10 aspect terms (listed in descending order of occurrence) that we identified in relation to various news headlines, as outlined in Table 2. These tables consistently highlight the usage of aspect terms such as *coronavirus*, *covid*, and *mask*, indicating their ongoing relevance throughout the year, while suggesting trending impacts throughout the year.

During the first quarter of the year, we observed frequent use of the terms *flights* and *passengers* around the same period as the first person-to-person transmission in late January. Upon further analysis of the associated tweets, it became evident that these terms were often accompanied by negative sentiment, expressing concerns about flight cancellations and the impact of the pandemic on the cruise line industry, leading to stranded passengers at sea^86^. Additionally, the term *china* emerged as a significant aspect term, but this pattern was observed only during the first quarter, suggesting a diminishing focus on China as a major factor related to the pandemic within the African American community.

Furthermore, the term *coronavirus* itself was the predominant aspect term throughout the first quarter. However, starting from March, it was gradually replaced by the term *covid* as the primary reference to the virus. Other terms such as *information*, *video*, and *agencies* were used in the context of information sharing, pandemic-related educational videos, and the role of government agencies in addressing the pandemic, respectively.

An intriguing observation during the first quarter of the year is the presence of the term *masks* as a frequent aspect term. This aligns with the period when debates about mask-wearing to mitigate the spread of the virus were ongoing, along with reports of mass production of masks^87–90^. However, as we moved into the second quarter, the context surrounding *mask(s)* shifted to discussions about the lack of mask-wearing in public or reservations about using masks on a daily basis. Most tweets expressing sentiments toward masks during the month of June were labeled as negative.

Another noteworthy finding is the emergence of the aspect term *pandemic*. Interestingly, despite WHO declaring COVID-19 as a pandemic on March 11, 2020^78^, this declaration did not seem to generate a peak in pandemic-related discourse on Twitter. However, the use of *pandemic* became more prominent in later peaks, particularly in June. The term *information* was used in a similar fashion as observed earlier in the year, while new terms such as *cases*, *care act*, and *court* emerged. These terms referred to the increasing number of positive COVID-19 cases, negative sentiments regarding changes related to the Affordable Care Act, and various decisions made by the U.S. Supreme Court during that period. Aspect terms *staff* and *place* were used in reference to hospital and office staff, as well as one’s home, likely indicating the impact of the pandemic on healthcare personnel and resources, as well as the transition to work-from-home practices.

Furthermore, a noteworthy finding is the use with the term *food*, which appeared in mid-June in discussions about food insecurities and healthy eating. It is worth noting that around this time, the U.S. food supply was adversely affected by the spread of the virus^91^. Overall, the second quarter of the year demonstrates an increased focus on mask-wearing and the recognition of the virus as a pandemic, along with concerns related to healthcare, including the food supply, which were not as prevalent at the beginning of the year.

During the summer months, the prominent aspect terms included *covid*, *masks*, *news*, and *pandemic* (see Table 4). In contrast, prior to this period, the top aspect terms exhibited more diversity, encompassing terms such as *coronavirus*, *covid*, *china*, *race*, *corona*, *news*, *information*, *thread*, *pandemic*, and *music*. This shift in focus during July indicates that the pandemic had become a central topic of discussion by that time. Other terms were utilized in distinct contexts, often conveying unfavorable experiences or opinions. For instance, the term *actor* emerged following the death of actor Nick Cordero due to COVID-19, while *churches* was associated with concerns about the potential spread of the virus upon the reopening of churches. The term *finger* arose from converting emojis to their textual equivalent and was utilized to express negative sentiment. Additionally, the term *doctor* was employed in reference to reports of healthcare professionals working long hours in hospitals^92^.

Table 5 also highlights the early emergence of the term *vaccine* in September, although it does not reappear as a frequent aspect term until mid-November. Notably, the term *intrusion* diverges from the prevailing trend of *covid* being the top aspect term used throughout September, as it reflects discussions regarding lockdown measures being viewed as an intrusion on human rights. Moving forward, Tables 6 and 7 demonstrate the continued usage of terms such as *mask*, *covid*, *pandemic*, and *news* until the end of the year. Additionally, terms like *court*, *vaccine*, and *bill* emerge during this period. References to vaccine production, particularly Pfizer-BioNTech’s vaccine, were prevalent. Furthermore, terms like *people*, *crew*, *staff*, *nurse*, *doctor*, and *woman* indicate a focus on how the pandemic was impacting people’s lives, potentially suggesting an empathetic perspective.

**Figure 7:**
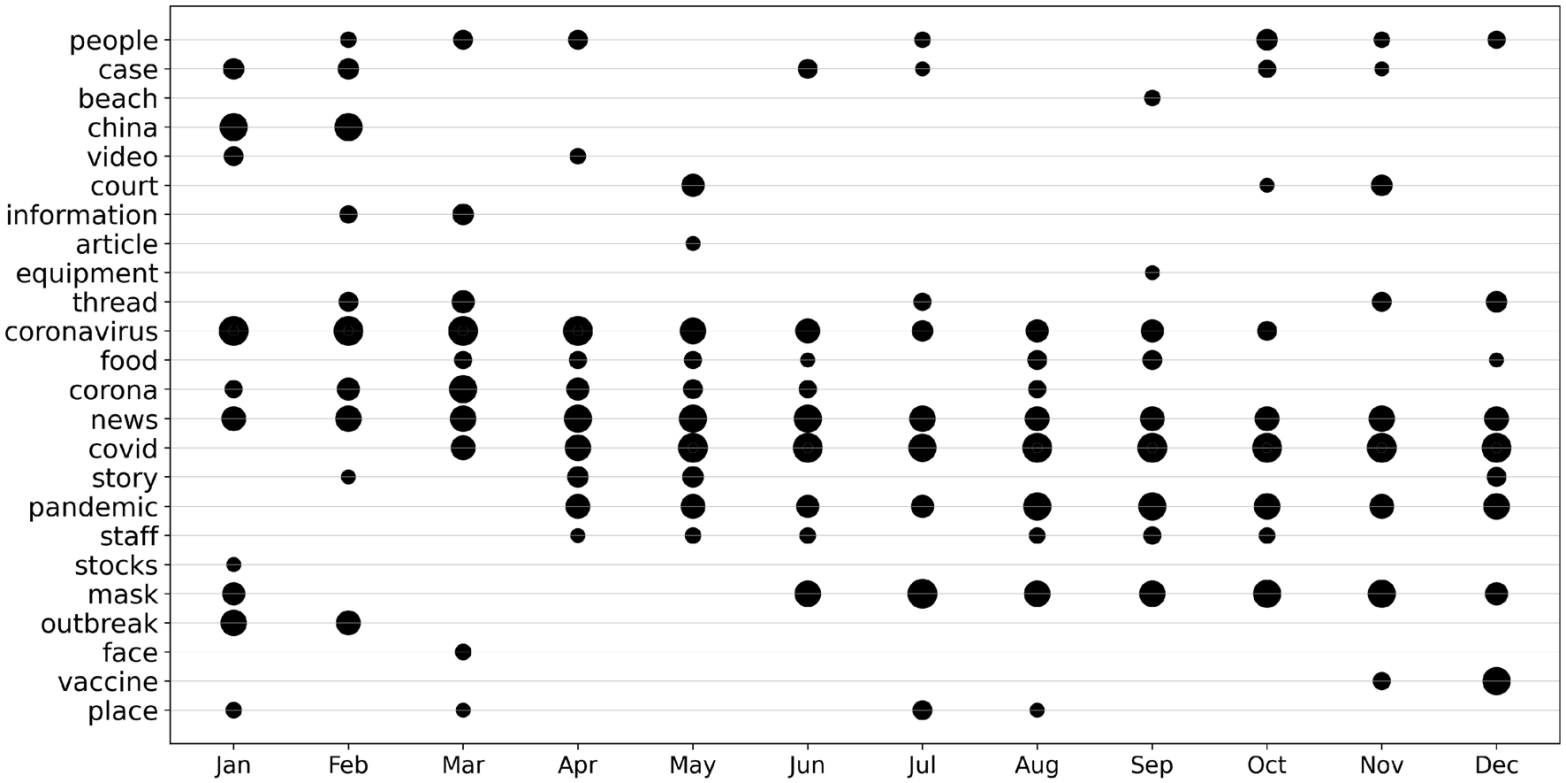
Use of prominent aspect terms over time.

Finally, the evolution of the top aspect terms’ frequency is illustrated in Fig. 7. The size of the markers corresponds to the frequency of the aspect term’s usage. For instance, the term *news* was consistently used throughout the year, while the use of *coronavirus* declined over time as *covid* gained prominence. Notably, the term *outbreak* ceases to appear once the pandemic is officially declared.

### Aspect terms and their relationships with semantically similar words

Tables 8 and 9 present the top 10 aspect terms per month in 2020, color-coded to indicate the aspect term. Each table includes five aspect terms, with 10 words listed beneath each of them. These 10 words were extracted from the Word2Vec model, a neural network model that learns an embedding for a given word given its surrounding words in a text sequence^93–96^, as semantically similar to the aspect term. These related words were used in similar contexts as the aspect term and thus have a degree of semantic similarity in the word embedding for a given month. These tables aim to uncover relationships between the most frequently used aspect terms and associated words, revealing insightful connections within the context of COVID-19-related tweets.

**Table 8:**
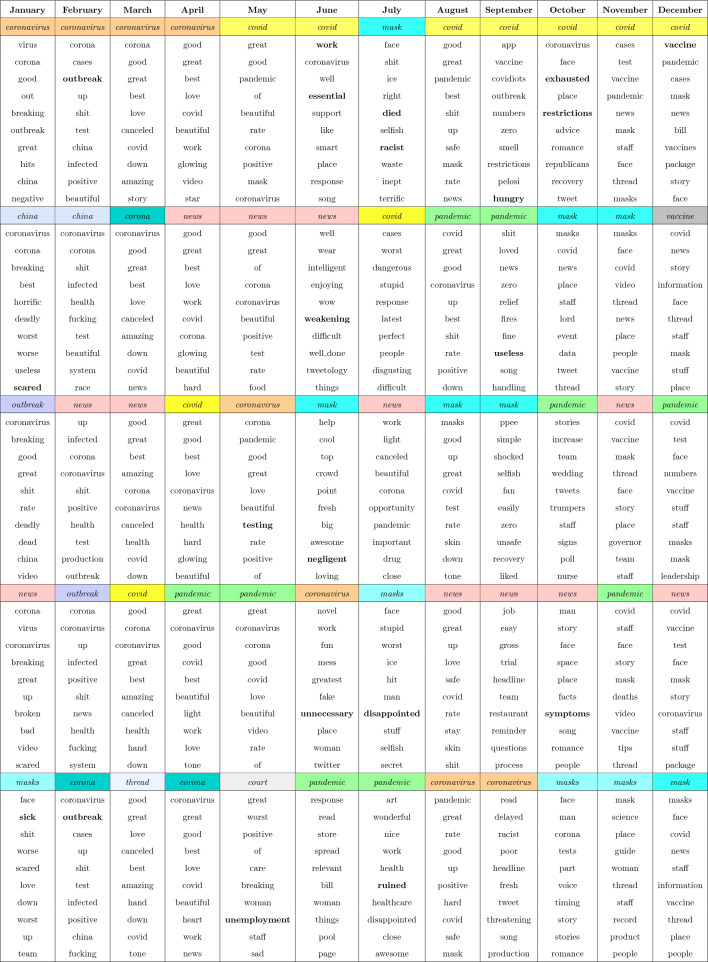
Top five aspect terms from January 2020 to December 2020 listing most similar words with respect to each aspect in decreasing order of occurrence.

**Table 9:**
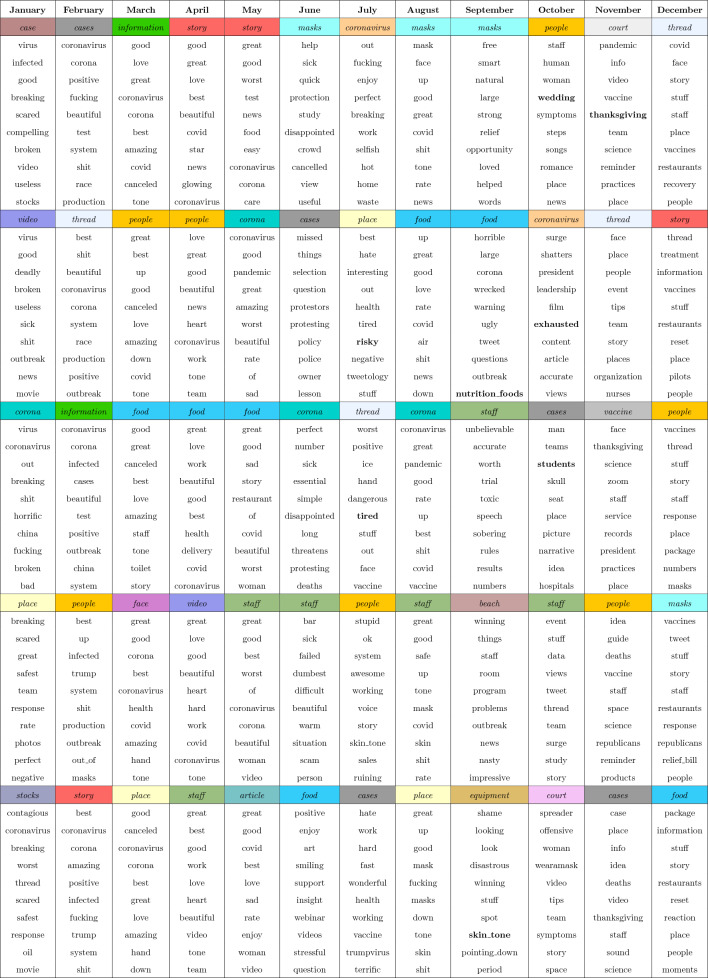
Next five aspect terms from January 2020 to December 2020 listing most similar words with respect to each aspect in decreasing order of occurrence.

For example, Table 8 indicates that the aspect term *coronavirus* was the most frequently occurring term from January to April. In February, words such as *outbreak*, *infected*, and *test* were also deemed semantically similar to *coronavirus*. This suggests that, according to Word2Vec, these words share a similar meaning or are commonly used in proximity to one another. That is, while *test* and *coronavirus* certainly have different definitions, their word embeddings are situated close to each other in the computational space. Consequently, the Word2Vec model’s findings provide valuable insights into words that are contextually related (i.e., frequently co-occurring). To emphasize these relationships, we have highlighted select words in bold. The intention behind this emphasis is to encourage readers to explore these intriguing connections. It is worth noting that the bolding is not intended to prioritize specific words but rather to draw attention to noteworthy relationships within the embedding space.

## Conclusion

Reports during the early stages of the COVID-19 spread in the United States drew attention to the disproportionately higher infection and death rates among African Americans. This underscores the importance of understanding the experiences and viewpoints of the African American community regarding the pandemic. Twitter data analysis has proven valuable in uncovering human behaviors and opinions across diverse domains. Thus, this study aimed to identify aspect terms in COVID-19-related tweets and examine their sentiment and temporal patterns, shedding light on how the pandemic has influenced the narratives of African Americans.

Specifically, we aimed to enhance our comprehension of the opinions and emotional responses among the African American population during the COVID-19 pandemic in 2020 by investigating patterns in Twitter data in relation to major news headlines in the United States. To achieve this, we constructed a robust machine learning pipeline comprising image and language-based classification models. The purpose of this pipeline was to filter out tweets that were unrelated to COVID-19 and tweets that were unlikely to have originated from a Twitter user identifying as African American. Subsequently, the filtered tweets were subjected to comprehensive aspect-based sentiment analysis.

Our findings indicate that a majority of tweets expressed negative sentiments, and the days with high tweet volumes appear to have coincided with significant U.S. events related to the pandemic, as evident from news headlines. The analysis revealed that commonly used aspect terms primarily revolved around the pandemic itself (e.g., *coronavirus*, *COVID*, *pandemic*). Further, initially, frequently used aspect terms focused on information sharing and the initial impacts of the pandemic, such as its effect on travel. As the year progressed, attention shifted towards topics like mask-wearing, recognition of the virus as a pandemic, and the government’s role in healthcare. The issue of food insecurity also garnered frequent conversations as the nation’s food supply strained during the second quarter of the year. In the later months, discussions surrounding vaccines became increasingly prevalent, along with tweets emphasizing the impact of the pandemic on the human population (e.g., *people*, *crew*, *staff*, or *nurse*).

Nevertheless, while our objective was to utilize Twitter data as a source of information about the pandemic’s impact on the U.S. African American population, it is crucial to acknowledge the limitations of this study. First, our analysis only encompasses the year 2020, warranting the need for future research to explore changes in opinions over time as the virus’s spread slowed in the U.S. Additionally, our aspect-based sentiment analysis relied on Microsoft Azure’s Cognitive Services, which solely identifies explicit aspect terms clearly specified by the author. We also observed challenges in cases where tweets employed words like “great,” as the sentiment could be misconstrued (e.g., use of the phrase “great concern,” for example, would likely lead to a positive sentiment). Furthermore, accurately classifying sentiments in tweets utilizing sarcasm proved to be a persistent challenge. We also note that the use of Twitter data alone may not provide a holistic view; additional data sources would be helpful for generalizing findings.

Thus, future investigations should prioritize the incorporation of implicit aspect extraction techniques to enhance the analysis, especially for tweets where the relevant terms are not explicitly stated. Additionally, expanding the scope of the study beyond 2020 and beyond the Twitter platform would yield valuable insights into the evolving opinions over time. Furthermore, it would be highly beneficial to compare our findings with those derived from other racial groups, enabling a more comprehensive understanding of the nuances and the generalizability of our current observations. It is important to note that we cannot definitively assert that our findings completely represent the targeted population, nor can we exclude the possibility of generalizability to other racial groups. These areas pose ongoing research challenges that necessitate further investigation and exploration.

Nevertheless, this research, focused on enhancing comprehension of African American concerns during the COVID-19 pandemic, has the potential to drive change in various areas. It could influence the way issues are approached, contribute to the addressing of health disparities, and bring attention to challenges specifically voiced by African Americans, including those that may not be widely acknowledged. By highlighting and amplifying these perspectives, this work can contribute to a more comprehensive understanding and pave the way for meaningful improvements and solutions.

## Supplementary Information

Supplementary Information.

## Data Availability

The datasets and code generated during and/or analysed during the current study are publicly available in the University of South Florida’s Department of Computer Science and Engineering’s Natural Language Processing Group’s Github repository: https://github.com/nlp-grp/Aspect_Based_Sentiment_Analysis_COVID19.
