# Supplementary material for: On the use of aspect-based sentiment analysis of Twitter data to explore the experiences of African Americans during COVID-19
Source: Sci Rep. 2023 Jul 2;13:10694. doi: 10.1038/s41598-023-37592-1 (PMC10315393; doi:10.1038/s41598-023-37592-1)
Supplement: Supplementary file 1 — Supplementary Information. [file 41598_2023_37592_MOESM1_ESM.pdf]

Appendix

A.1 Survey Questions Used for Classifying Twitter Data

1. Tell us a bit about how COVID-19 has impacted you.

2. In the situations when you have to leave your home, what do you usually do to protect yourself and those around you from COVID-19?

3. If needed, are you willing to seek help from local officials or medical facilities because of COVID-19? Why or why not?

4. What problems or barriers do you believe you may face should you decide to seek help?

5. Since the onset of COVID-19, do you feel that you have experienced any unfair treatment when trying to access resources such as medical care, education, work opportunities, or other services? If so, why do you think that is?

6. Do you feel that the incident(s) you are referring to has impacted your perception or decision-making during the current COVID-19 pandemic, and if so, in what ways?

7. Please read the following articles or tweets and provide a review of their contents. For example, do you relate to any of the content? Do you find the article to be true or false? Have you experienced any of the incidents reflected in the article? What is your overall opinion of the article?

   (a) **44% of Low-Income Americans are Using Savings or Retirement Money to Pay Bills**

   A new survey from the Pew Research Center finds that 44% of lower-income people say they have dipped into their retirement or other savings since the start of the coronavirus crisis, while only 16% of upper-income people say they have done so. Overall, a third of Americans say they have turned to savings or retirement accounts to pay the bills since the pandemic broke out in February. Just as with so many other aspects of this crisis, the disparity found in Pew's survey, which polled 13,200 US adults over two weeks in August, falls in large part along racial lines.

   The cruel irony, of course, is that the groups most likely to dip into savings to stay afloat are less likely to have savings or retirements to draw from. In an earlier Pew study from April, 53% of white adults said they had rainy day funds, while less than a third of Hispanic, Black, and low-income adults said the same. Meanwhile, the new Pew survey indicates that half of all US adults who lost a job in the coronavirus crisis continue to be unemployed. "[W]e're seeing something similar where those who are lower income and lower wealth to begin with, they fall further down," Kochhar says.

Original Publication: *Cheng, M. (2020, October 1). 44% of low-income Americans are using savings or retirement money to pay bills. Quartz. https://qz.com/1908543/33-percent-in-the-us-are-dipping-into-savings-or-retirement-to-pay-bills*

(b) **Researchers Find Doubts About COVID-19 Vaccine Among People Of Color**

At a meeting experts advising the FDA on COVID-19 vaccines, the concerns of front-line workers and people of color were read aloud verbatim, highlighting the crucial project of communicating the safety and effectiveness of a vaccine in an environment of deep political distrust. Those concerns were gathered at a series of listening sessions organized by the Reagan-Udall Foundation, a nonprofit that aims to advance the work of the FDA. Participants in the sessions voiced a range of concerns:

- "I would not be first in line and I would want to see some data."
- "When I hear the FDA say they have a particular process, but then I hear the White House say they can cut it in half or negate it – it brings more distrust."
- "We are not going to be guinea pigs again." "African Americans are treated differently by doctors."
- "I am looking for an organization I can trust that does not have a tainted history and has not been bought out by some big pharma."

The listening sessions focused on two groups. One was front-line workers in service, retail and health care settings. The second focused on people who are often underrepresented and are at increased risk for COVID-19: Black, Hispanic and Indigenous/Native American communities. The foundation said its goal is to understand the perceptions that may lead Americans to feel hesitant about receiving a COVID-19 vaccine – and to use that information to craft messaging that addresses those concerns. Winckler noted that the participants' concerns often shared certain themes: concerns about the speed of the process, distrust of government and government agencies, distrust of the health care system, and concern that politics and economics will be prioritized over science. People of color also voiced worries that the vaccine won't work for minority populations. Among their statements:

- "Need to know other minorities have taken it. Are other minorities ok? We're all built different. How do we know?"
- "I need to know that all the minorities who took it are okay. I need to know it works for everybody. I am not trying to be harmed."

Original Publication: *Wamsley, L. (2020, October 22). Researchers Find Doubts About COVID-19 Vaccine Among People Of Color. NPR. https://www.npr.org/sections/coronavirus-live-updates/2020/10/22/926813331/researchers-find-doubts-about-covid-19-vaccine-among-people-of-color*

(c) **Another Surge, Vaccine and Treatment Progress, and More Coronavirus News**

What we know about coronavirus as cases climb once again

This week the virus continued to surge throughout much of the United States and Europe. Stateside, it was the first day with more than 70,000 new cases since the summer, as 32

states reported rising rates of infection. While the subject of how and whether to reopen schools remains controversial, experts say that so far there's little to suggest that reopened schools are contributing to these surges. And though infections are rising, Covid-19 death rates have plummeted, an indication that doctors are getting better at caring for patients even in the absence of a definitive cure or treatment.

A vaccine advances as America's first treatment is greenlit

Yesterday, Moderna announced that it had enrolled all 30,000 participants in its Phase III vaccine trial. More than a third are minorities and a quarter are over the age of 65 in an attempt to reflect the diversity of the general population. The company expects to have early data about the efficacy of its vaccine in the next month. During a daylong meeting with FDA officials the same day, some vaccine experts urged the agency to request more than the requisite two months of safety data before approving vaccines. The FDA has also moved forward with remdesivir as the first—and only—fully approved drug for treating Covid-19 in the US. Now, it can be used for any hospitalized Covid-19 patient in the US who's at least 12 years old. This news comes approximately a week after a massive international trial found that remdesivir does not prevent deaths among patients with severe cases with Covid-19. The research has not yet been peer reviewed or published in a journal, and some have disputed its conclusiveness.

New measures are taken to curb the viral spread of pandemic misinformation

In the hopes of curbing the rampant spread of coronavirus misinformation, the WHO announced yesterday that it will grant Wikipedia free use of its published information, graphics, and videos in a first-of-its-kind collaboration. The WHO translates its work into six official languages, whereas Wikipedia content is available in around 175. Since the start of the pandemic, Wikipedia—once widely regarded as unreliable—has been a key resource for debunking misinformation about coronavirus.

Original Publication: *Sneider, E. (2020, October 23). Another Surge, Vaccine and Treatment Progress, and More Coronavirus News. WIRED. https://www.wired.com/story/another-surge-vaccine-and-treatment-progress-and-more-coronavirus-news/*

(d) "For goodness sake. Covid has no solution at present." (Extracted from Twitter)

(e) "found that employed adults who tested positive for #COVID19 were more likely to report going to work/school in person" (Extracted from Twitter)
